# A testis-specific lncRNA functions as a post-transcriptional regulator of *MDM2* and stimulates apoptosis of testicular germ cell tumor cells

**DOI:** 10.1038/s41420-024-02119-8

**Published:** 2024-08-03

**Authors:** Saya Ito, Akihisa Ueno, Takashi Ueda, Ryota Ogura, Satoshi Sako, Yusuke Gabata, Junki Murashita, Hikaru Takahashi, Osamu Ukimura

**Affiliations:** Department of Urology, Graduate School of Medical Science, Kyoto Prefectural University of Medicine, Kyoto-City, Kyoto Japan

**Keywords:** Non-coding RNAs, Testicular cancer

## Abstract

Germ cells preferentially induce apoptosis in response to DNA damage to avoid genomic mutations. Apoptosis of germ cells is closely related to cancer development and chemotherapy resistance; however, its regulatory mechanism is unclear. Here, we suggest that testis-specific lncRNA *LINC03074* is involved in male germ cell apoptosis by regulating the expression of the proto-oncogene *MDM2*. *LINC03074* is highly expressed in the sperm of healthy adult testes and cancer cells of testes with testicular germ cell tumors (TGCTs). *LINC03074* binds to *MDM2* mRNA via an *Alu* element, thereby reducing MDM2 protein levels. *LINC03074* stimulates STAU1-mediated nuclear export of *MDM2* mRNA by increasing STAU1 binding to *MDM2* mRNA in the cell nucleus, thereby promoting PKR-mediated translational repression in the cytoplasm. The induction of apoptosis in TGCT cells and their responsiveness to the anticancer drug cisplatin is enhanced by *LINC03074*. Notably, *LINC03074* increased E2F1 expression without increasing p53, the primary target of MDM2, and upregulated the apoptotic gene *p73*, the target gene of E2F1. *LINC03074*-mediated regulation of apoptosis contributes to the responsiveness of TGCTs to anticancer drug-induced DNA damage.

## Introduction

Testicular germ cell tumors (TGCTs) are generally highly sensitive to platinum-based chemotherapy, such as cisplatin, but some are chemotherapy-resistant [1]. Mouse double minute 2 (MDM2) is amplified in various human malignancies, including TGCTs, and MDM2 overexpression is associated with chemotherapy resistance [2, 3]. MDM2 is a major negative regulator of p53, promoting ubiquitin-dependent proteasomal degradation of p53 as an E3 ubiquitin ligase and repressing p53 transcriptional activation [4, 5]. The tumor suppressor p53 is commonly mutated in various types of cancers [6, 7], whereas it is often overexpressed and rarely mutated in TGCTs [3]. Accordingly, MDM2 overexpression in TGCTs is predicted to affect the activity of wild-type p53; however, the molecular mechanisms underlying the sensitivity or resistance of TGCTs to chemotherapy remain unclear.

*MDM2* expression and activity are regulated at multiple levels, from transcription to numerous post-translational modifications, in addition to genomic alterations, such as copy number variations, mutations, and polymorphisms [8]. Post-transcriptional regulation of *MDM2* at the mRNA level has been widely reported to modulate transcript stability via miRNAs [9]. Human *MDM2* has a very long 3′UTR (~5.7 kb) that retains a multitude of potential miRNA targets [10]. Notably, *MDM2* contains multiple transposable elements containing *Alu* in the 3′UTR [11]. *Alu* elements are abundant retrotransposon elements that spread throughout the human genome and occupy a significant portion of the 3′UTR [12]. The inverted repeat *Alu* element (IR*Alus*) in the 3′UTR forms a double-stranded RNA (dsRNA) structure, which serves as a target for dsRNA-binding factors such as Staufen1 (STAU1) and adenosine deaminase acting on RNA (ADAR1) [13, 14]. STAU1 binds to IR*Alu* in the 3′UTR to undergo various RNA metabolic processes, such as RNA synthesis, folding, modification, processing, translation, and decay [13]. ADAR1, an adenosine-to-inosine (A-to-I) RNA-editing enzyme, competes with STAU1 for the occupancy of target RNAs, thereby inhibiting STAU1-mediated nuclear retention or decay of RNAs [15, 16]. In *MDM2* gene expression, STAU1 and ADAR1 appear to mediate post-transcriptional regulation via binding to the IR*Alus* of the *MDM2* 3′UTR, a mechanism independent of alterations in mRNA stability and miRNA targeting [16].

Here, we show the role of *LINC03074*, a testis-specific lncRNA with an *Alu* element, in TGCT cells. *LINC03074* binds to *MDM2* mRNA via *Alu*, thereby stimulating STAU1-mediated nuclear export of *MDM2* mRNA. Consequently, MDM2 is reduced by PKR-mediated translational repression, which in turn promotes the apoptosis of TGCT cells. Our findings provide molecular mechanistic insights into the drug responsiveness of TGCTs by demonstrating a regulatory mechanism of apoptosis in TGCT cells.

## Results

### *LINC03074* shows different expression patterns between cancerous and normal sperm

**Fig. 1:**
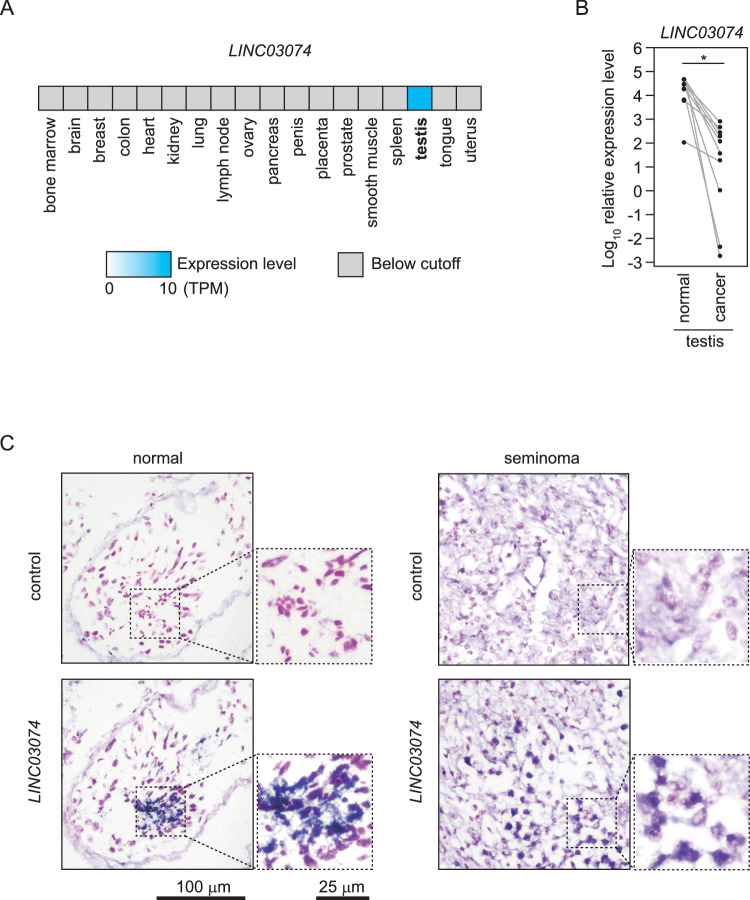
Expression of *LINC03074* in testis. **A** Expression of *LINC03074* in major human tissues. The data resulted from an RNA-Seq CAGE analysis of human tissues of the RIKEN FANTOM5 project. Expression levels in tissues other than testis are less than 0.5 TPM, which is below the detection limit of the analysis. **B** *LINC03074* levels in normal and cancer regions of testis tissues from patients with seminoma. Paired normal and cancer tissue samples were obtained from 10 different testicles (paired dots are connected by gray lines). Relative expression levels of *LINC03074* to *GAPDH* were measured using RT-qPCR. *P = 0.00178. **C** In situ hybridization with detection probes for *LINC03074* on testes of healthy adults and of patients with seminoma. The sense strand probe of *LINC03074* was used as a negative control. The right small panels show enlarged views of the corresponding left panels. *LINC03074* was localized to the nucleus and cytoplasm in normal sperm cells and mainly to the nucleus in seminoma cells.

To elucidate the characteristics of TGCTs, we compared the gene expression profiles of cancer tissues from seminoma patients with those of matched normal adjacent tissues [17]. A total of 565 genes, among the 50,599 genes tested, exhibited a more than 2-fold increase in RNA expression in seminoma tissues compared to normal adjacent tissues, including 18 genes encoding lncRNAs (data not shown). In contrast, 431 genes exhibited a >2-fold decrease in expression in cancer tissues, including 59 genes encoding lncRNAs (data not shown). To identify the lncRNA that determines the characteristics of testicular tumor cells, we focused on *LINC03074* (*LOC100505685*), which showed marked differences in expression between cancerous and normal tissues. According to the database, *LINC03074* is expressed specifically in the testes of humans (Fig. 1A). Moreover, a recent study identified *LINC03074* as a testis-specific lncRNA [18]. The expression pattern of *LINC03074*, which was significantly higher in normal tissues than in cancerous tissues of the testes of patients with seminoma, was confirmed via relative quantitative analysis using RT-qPCR (*P* = 0.00178, Fig. 1B). We performed ISH with a *LINC03074* detection probe in the testes of healthy adults and patients with seminomas. The results revealed that *LINC03074* was localized to the nucleus and cytoplasm of normal spermatids, whereas it was mainly localized to the nucleus of seminoma cells (Fig. 1C). We further quantified the expression of *LINC03074* in four types of cultured cells derived from seminoma and non-seminoma tissues and found that its expression was significantly higher in TCam-2 seminoma cells (Supplementary Fig. S1) [19]. These results suggest that *LINC03074* functions in both testis-derived seminoma cells and normal cells, although it is differentially expressed in cancer and normal cells.

### *LINC03074* interacts with *MDM2* mRNA via *Alu* element

**Fig. 2:**
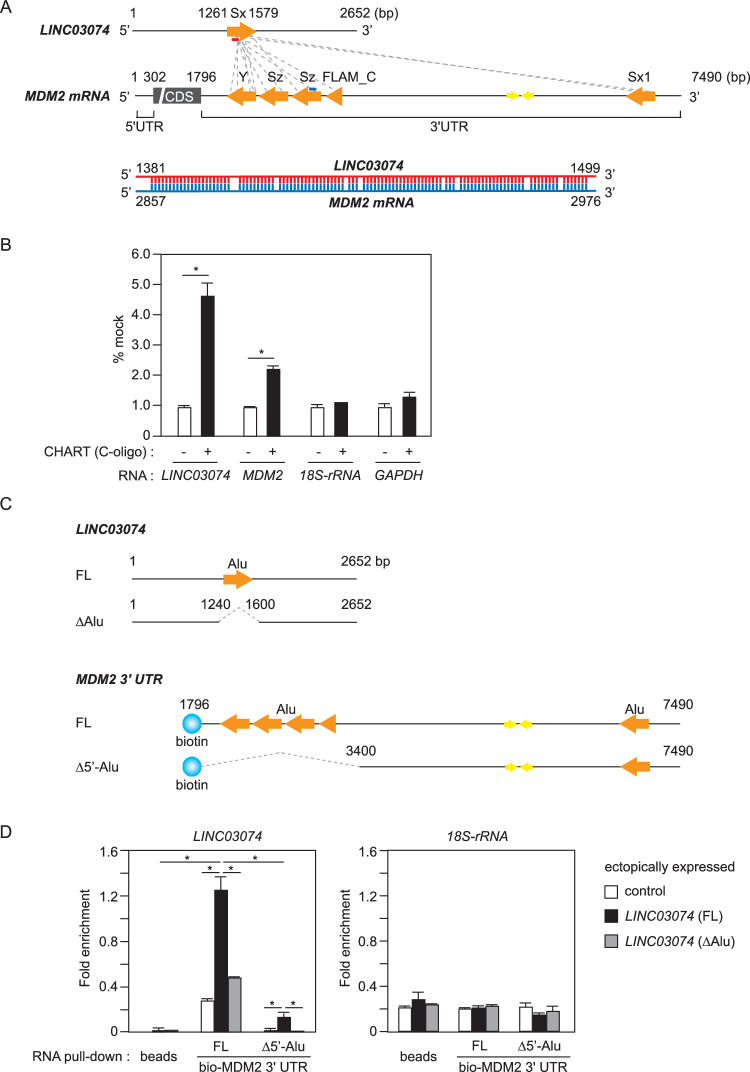
Interaction of *LINC03074* with *MDM2* mRNA. **A** Schematic drawing of predicted interaction regions between human *LINC03074* and *MDM2 mRNA*. Interaction regions predicted by the lncRRIsearch database (gray dot lines), *Alu* elements (orange arrows), and inverted *Alu* pairs (yellow arrows) are shown (upper panel). An example of the predicted base pairs for the regions shown in red and blue in the upper panel is shown (ΔG = −60.91 kcal/mol, bottom panel). Sx, Sx1, FLAM_C, Sz and Y, *Alu* subfamilies; 5′UTR 5′untranslated region, CDS coding sequence, 3′UTR 3′untranslated region. **B** Enrichment of RNAs by *LINC03074* CHART as measured using RT-qPCR. Each enrichment value is shown as a percentage of the measurement for each mock (without C-oligo). Error bars represent +SEM for three qPCR experiments. *P < 0.05. **C** Schematic representation of full length (FL) and *Alu* element-deficient (ΔAlu) *LINC03074* and biotin-tagged *MDM2* 3′UTR (FL and Δ5′-Alu). **D** RNA pull-down assays using the biotin-tagged (bio)-*MDM2* 3′UTR. In vitro, transcribed bio-*MDM2* was incubated with total RNA extracted from HEK293 cells overexpressing *LINC03074*. RNAs associated with bio-*MDM2* 3′UTRs were detected via RT-qPCR. Error bars represent +SEM for three qPCR experiments. *P < 0.05.

To elucidate the function of *LINC03074* in testicular cells, we searched databases to identify the elements with which this lncRNA could interact. Using lncRRIsearch, *MDM2* mRNA was identified as a candidate interacting factor for *LINC03074* [20]. *LINC03074* contained one *Alu* element, while *MDM2* mRNA contained five *Alu* elements and a pair of inverted-*Alu*s in the 3′UTR (Fig. 2A). There were multiple candidate sequences for the interaction between *LINC03074* and *MDM2* mRNA within each *Alu* element (Fig. 2A). The sequences of *LINC03074* and the sense strand of *MDM2* mRNA were nearly complementary, as shown in an example of a candidate interaction region (ΔG = −60.91 kcal/mol, lower part of Fig. 2A). We decided to use TCam-2 cells as a model for seminoma cells in the following experiments, considering that *LINC03074* is likely to function in seminomas according to its expression pattern (Fig. 1C and Supplementary Fig. S1). To determine whether *LINC03074* binds to *MDM2* mRNA, we performed CHART assays using TCam-2 cells. CHART enables the identification of associated targets of lncRNAs by enriching lncRNAs with their targets using affinity-tagged oligonucleotides (C-oligo) to capture endogenous lncRNAs in cross-linked cell extracts [21]. *LINC03074* was enriched in TCam-2 cell extracts by CHART using a C-oligo for *LINC03074* (Fig. 2B). We then tested whether mRNAs of *MDM2*, *18S-rRNA* and *GAPDH* were enriched using *LINC03074* CHART, and found that *MDM2* mRNA was associated with *LINC03074* (Fig. 2B). Approximate estimates using qPCR showed that the molecular ratio of *LINC03074* to *MDM2* mRNA in TCam-2 cells was approximately 1:166 (Supplementary Fig. S2). To confirm whether *LINC03074* and *MDM2* mRNA interact via their respective *Alu* elements, we generated *Alu* element-deficient *LINC03074* and *MDM2* expression constructs (Fig. 2C). We performed an RNA pull-down assay with the 3′UTR region of biotin-labeled *MDM2* mRNA using total RNA extracted from HEK293 cells transiently expressing *LINC03074*. We found that *LINC03074* (FL) binds to the *MDM2* 3′UTR (FL) (Fig. 2D). In comparison, *LINC03074* (FL) binds to the *MDM2* 3′UTR (Δ5′-Alu) was attenuated (Fig. 2D). In addition, *LINC03074* (ΔAlu) showed significantly weaker binding to *MDM2* than *LINC03074* (FL) (Fig. 2D). In contrast, no binding was detected between *18S-rRNA* and *MDM2* 3′UTR (either FL or Δ5′-Alu) (Fig. 2D). These results indicated that *LINC03074* binds to the *Alu* elements of *MDM2* mRNA via its own *Alu* element.

### *LINC03074* inhibits *MDM2* gene expression by binding to *MDM2* mRNA

**Fig. 3:**
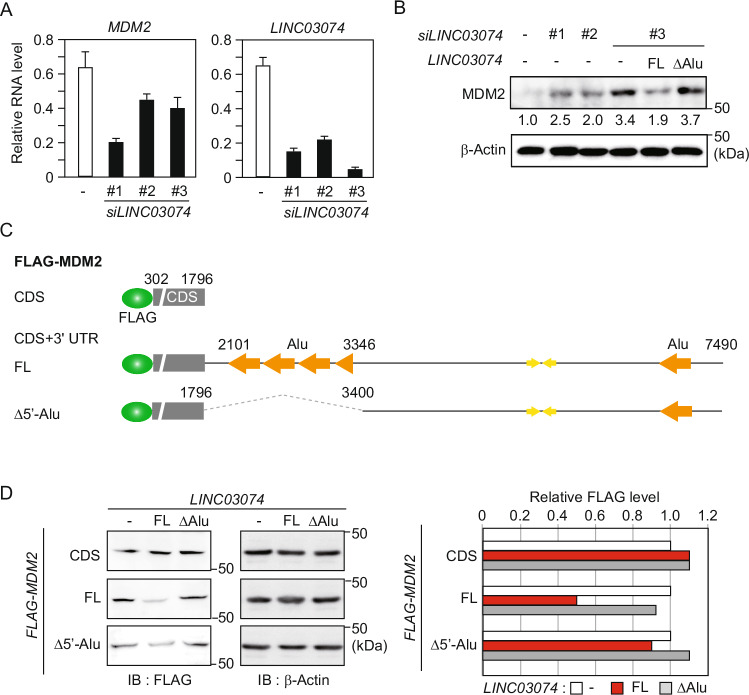
Reduction of MDM2 protein levels by *LINC03074.* **A** *MDM2* and *LINC03074* RNA levels in *LINC03074*-knockdown TCam-2 cells measured via RT-qPCR. TCam-2 cells were transfected with siRNAs for *LINC03074* (*siLINC03074*) for 72 h. Data represent the average of three independent measurements normalized to *GAPDH* mRNA expression. **B** MDM2 protein levels in *LINC03074* knockdown cells transiently expressing the *LINC03074* mutant. Western blotting was performed with anti-MDM2 antibody using TCam-2 cells transfected with *siLINC03074* for 48 h followed by *LINC03074* expression plasmids for 24 h. Band intensity was quantified by Image Lab 6.1. The measurements were normalized to the *siControl* protein levels that are indicated at the bottom of each band. **C** Schematic representation of the constructs containing FLAG tag-fused *MDM2* CDS alone or in combination with 3′UTR. **D** Western blot analysis using an anti-FLAG antibody against HEK293 cells transfected with FLAG-tagged MDM2 and *LINC03074* expression plasmids (left panels). The relative intensity to the band of the control in each first left lane is shown in the bar graph (right panel).

We investigated the effects of the interaction of *LINC03074* with *MDM2* mRNA on the expression of the *MDM2* gene. To examine the effect of *LINC03074* on mRNA stability, we quantified *MDM2* mRNA levels following knockdown of *LINC03074* using three different siRNAs, and found a decrease in mRNA levels for all siRNAs (Fig. 3A). In contrast, MDM2 protein levels were increased by *LINC03074* knockdown (Fig. 3B). The increase in MDM2 protein by *LINC03074* knockdown was rescued by the transient expression of full-length *LINC03074* (FL), but not by the ΔAlu mutant (ΔAlu) (Fig. 3B). Next, we verified that *LINC03074*-induced alterations in MDM2 protein levels were caused by the binding of *LINC03074* to *MDM2* mRNA. Flag tag fusion MDM2 protein expression plasmids were generated by inserting 3′UTR sequences (FL or Δ5′-Alu) downstream of the CDS of *MDM2* (Fig. 3C). These plasmids were transfected together with *LINC03074* (FL or ΔAlu) expression plasmids into HEK293 cells, and the protein levels of FLAG-MDM2 were quantified by immunoblotting using an anti-FLAG antibody. In the absence of the 3′UTR, FLAG-MDM2 levels remained unchanged regardless of the presence of *LINC03074* (Fig. 3D). However, in the presence of the 3′UTR (FL), FLAG-MDM2 levels were significantly reduced by *LINC03074* (FL), but not by *LINC03074* (ΔAlu) (Fig. 3D). In the case of *Alu* elements in the 3′UTR were absent (Δ5′-Alu), FLAG-MDM2 levels were only slightly reduced by *LINC03074* (Fig. 3D). These findings suggest that *LINC03074* binding to *MDM2* mRNA via *Alu* elements may influence the post-transcriptional or translational processes of *MDM2* gene expression.

### *LINC03074* enhances STAU1-mediated nuclear export and PKR-induced translational repression of *MDM2* mRNA

**Fig. 4:**
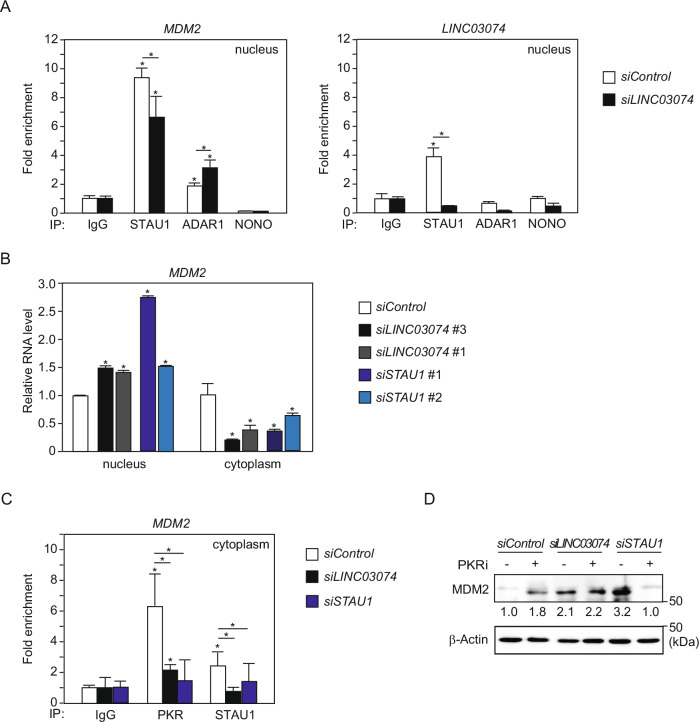
Decrease of *LINC03074* suppresses STAU1-mediated mRNA nuclear export and PKR-induced translational repression of *MDM2.* **A** Effect of *LINC03074* on the binding of dsRNA binding proteins and *MDM2* mRNA in TCam-2 cell nuclei. RIP assay was performed with each dsRNA binding protein antibody using TCam-2 cell nuclear extracts with *LINC03074* knockdown. The level of RNA binding with each dsRNA binding protein was measured via RT-qPCR and is shown as a relative value to the IgG binding level. Error bars represent +SEM for three qPCR experiments. An asterisk above each bar indicates statistical significance for IgG values. *P < 0.05. **B** Effect of *LINC03074* on *MDM2* mRNA levels in the nucleus and cytoplasm of TCam-2 cells. The relative expression of *MDM2* to *5S-rRNA* (nucleus) or *GAPDH* (cytoplasm) was measured using RT-qPCR. Error bars represent +SEM for three qPCR experiments. An asterisk above each bar indicates statistical significance for *siControl* values. *P < 0.05. **C** RIP assay using anti-PKR and anti-STAU1 antibodies with cytoplasmic extracts from TCam-2 with knockdown of *LINC03074* or STAU1. Error bars represent +SEM for three qPCR experiments. An asterisk above each bar indicates statistical significance for IgG values. *P < 0.05. **D** Western blotting of *LINC03074* or STAU1 knocked-down TCam-2 cells treated with PKR inhibitor. TCam-2 cells were transfected with *siLINC03074* and siSTAU1 for 24 h, followed by treatment with 1μM PKR inhibitor for 24 h. Band intensity was quantified by Image Lab 6.1. The measurements were normalized to control (*siControl* without PKR inhibitor) protein levels that are indicated at the bottom of each band.

It has been reported that the inverted repeat *Alu* elements (IR*Alus*) in the 3′UTR of mRNA serve as a binding site for ADAR1, a dsRNA-specific enzyme that performs A-to-I RNA editing [16]. The mRNA with 3′UTR IR*Alus* edited by ADAR1 is retained in the nucleus through interaction with paraspeckle, which is formed by the nuclear lncRNA, *NEAT1*, and its binding partner, NONO [22]. Alternatively, IR*Alus* in the 3′UTR of mRNA binds to the dsRNA-binding protein STAU1, which is involved in various RNA metabolic regulations [13]. STAU1 binds to IR*Alu* to facilitate the export of IR*Alu* mRNA from the nucleus to the cytoplasm, while competitive inhibition of NONO binding to IR*Alu* prevents NONO-mediated mRNA retention in the nucleus [23]. We examined the binding of *MDM2* mRNA to STAU1, ADAR1, or NONO in the nuclei of TCam-2 cells and the effect of *LINC03074* on their binding. RIP assays using nuclear extracts of TCam-2 cells showed that *MDM2* mRNA binds to STAU1 and ADAR1, but not NONO (Fig. 4A). Knockdown of *LINC03074* suppressed the binding of *MDM2* mRNA to STAU1 while increasing its binding to ADAR1 (Fig. 4A). In contrast, *LINC03074* bound only to STAU1 but not to ADAR1 and NONO (Fig. 4A). Considering that STAU1 is responsible for RNA shuttling, we investigated whether *LINC03074* affected the nuclear export of *MDM2* mRNA. *MDM2* mRNA was increased in the nucleus and decreased in the cytoplasm following *LINC03074* and STAU1 knockdown (Fig. 4B). These results suggested that *LINC03074* promotes the recruitment of STAU1 to *MDM2* mRNA in the nucleus and facilitates STAU1-mediated nuclear export.

Taken together, the downregulation of *LINC03074* increased intracellular MDM2 protein levels, despite decreasing *MDM2* mRNA levels in the cytoplasm (Figs. 3B and 4B). We speculated that *LINC03074*-mediated enhancement of STAU1 and *MDM2* mRNA interactions in the nucleus leads to the translational repression of *MDM2*. STAU1 binding to IR*Alu* mRNA promotes nuclear export and translation [23]. However, STAU1-mediated mRNA nuclear export is promoted when the paraspeckle component is downregulated, whereas protein kinase R (PKR)-mediated translational repression in the cytoplasm is promoted [23]. PKR is activated by binding to virus-derived dsRNA and phosphorylates eukaryotic translation initiation factor 2A (eIF2a), resulting in translational inhibition [24, 25]. To determine whether PKR can bind to *MDM2* mRNA in TGCT cells, we performed RIP assays with cytoplasmic extracts from TCam-2 cells using a PKR antibody. PKR was found to bind to *MDM2* mRNA in the cytoplasm, and this interaction was reduced by *LINC03074* and STAU1 knockdown (Fig. 4C). Furthermore, STAU1 and *MDM2* mRNA binding in the cytoplasm was attenuated by *LINC03074* and STAU1 knockdown (Fig. 4C). Finally, we determined whether *MDM2* was translationally repressed by PKR activation. MDM2 protein levels were increased by PKR inhibitor treatment of TCam-2 cells (Fig. 4D). The increase in MDM2 protein expression induced by PKR inhibitors was not detected with *LINC03074* and STAU1 knockdown (Fig. 4D). These results indicated that *LINC03074*, similar to paraspeckle components, modulates the nuclear export of STAU1-bound *MDM2* mRNA, thereby facilitating PKR-mediated translational repression.

### *LINC03074* enhances cisplatin-induced apoptosis and cell growth inhibition

**Fig. 5:**
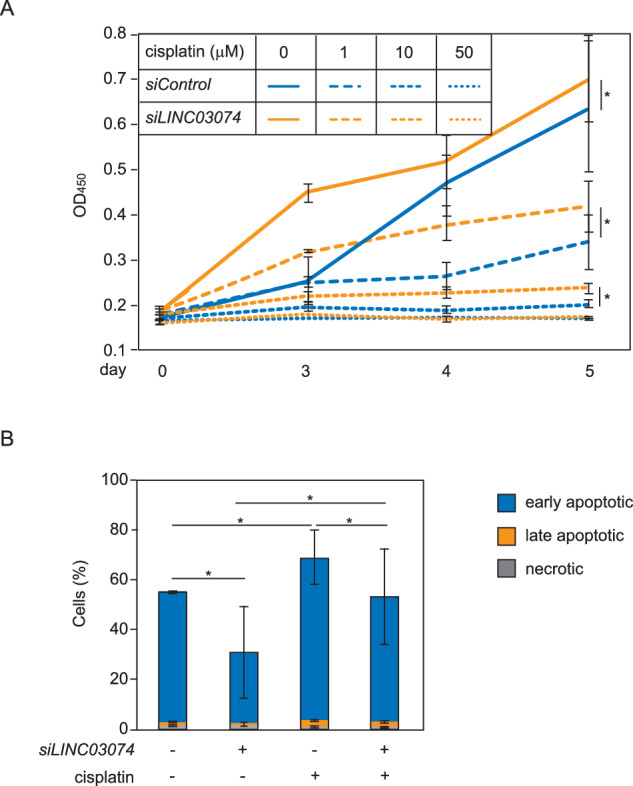
*LINC03074* enhances cisplatin-induced apoptosis. **A** Cell growth assay using TCam-2 cells transfected with *siLINC03074* and treated with different concentrations of cisplatin. Absorbance at 450 nm (OD_450_) was used to estimate cell concentration. Data represent the means ± SEM (*n* = 3). **B** Apoptosis assay using *LINC03074-*knockdown TCam-2 cells treated with cisplatin. TCam-2 cells were transfected with *siLINC03074* and treated with 20 μM cisplatin for 48 h. Apoptotic cells were identified by the increase in the fluorescence intensity of FITC-labeled Annexin-V using flow cytometry. Percentage of TCam-2 cells (either with or without *siLINC03074*) in apoptosis in the presence or absence of cisplatin (*n* = 3). *P < 0.05. *siControl*, TCam-2 cells transfected with a negative control siRNA.

To assess whether *LINC03074* affects the proliferation of TGCT cells, CCK8 analysis was performed using cisplatin, a platinum chemotherapeutic agent that induces DNA damage in cancer cells by inhibiting DNA repair [26]. The growth of TCam-2 cells was inhibited by cisplatin treatment in a concentration-dependent manner (Fig. 5A). *LINC03074* knockdown enhanced the growth of TCam-2 cells and attenuated cisplatin-induced inhibition of cell growth (Fig. 5A). In contrast, *LINC03074* knockdown had no effect on the growth of non-seminoma-derived NEC8 cells expressing low *LINC03074* levels (Supplementary Fig. S3). First, the effect of *LINC03074* on the cell cycle was assessed; however, no cell cycle abnormalities due to *LINC03074* knockdown or cisplatin treatment were observed under the conditions examined (Supplementary Fig. S4). Next, the effect of *LINC03074* on apoptosis was examined using FACS analysis. *LINC03074* knockdown reduced the frequency of spontaneous and cisplatin-induced apoptosis (Fig. 5B and Supplementary Fig. S5). These results indicate that *LINC03074* inhibits the proliferation and promotes the apoptosis of seminoma cells. Furthermore, the responsiveness of seminoma cells to cisplatin-induced DNA damage was enhanced by *LINC03074*.

### *LINC03074* increases E2F1 levels and upregulates *p73* gene expression

**Fig. 6:**
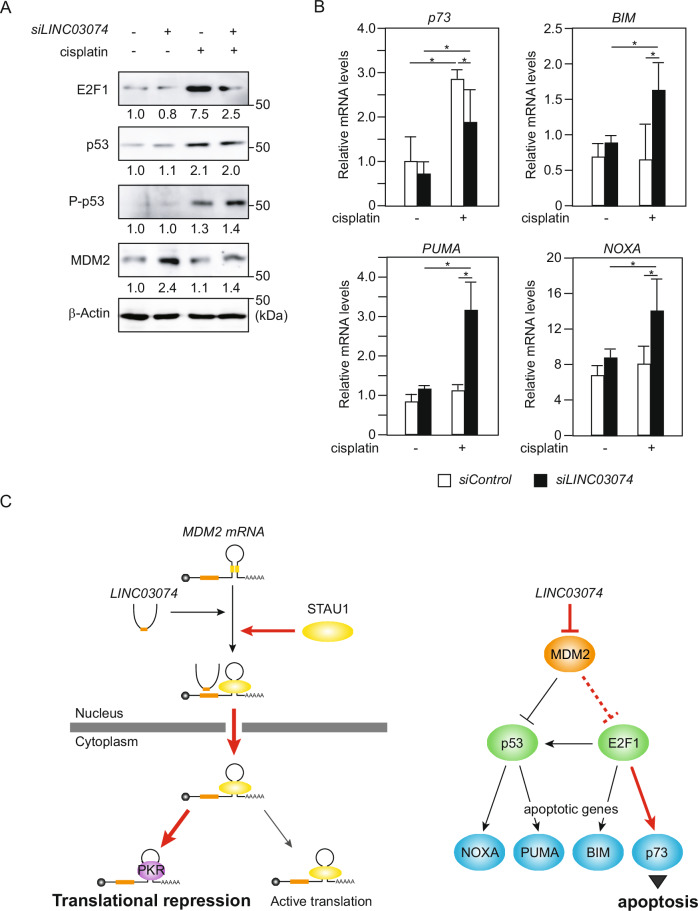
*LINC03074* activates E2F1 and p73 pathways. **A** Western blotting using TCam-2 cells transfected with *siLINC03074* for 72 h. Cisplatin was added to the culture medium at a concentration of 10 μM for 72 h before cell extraction. Band intensity was quantified using Image Lab 6.1, and all measurements were normalized to the protein levels of the *siControl* without cisplatin (indicated at the bottom of each band). **B** Relative expression levels of apoptotic genes in *LINC03074*-knockdown cells were measured using RT-qPCR. TCam-2 cells were transfected with *siLINC03074* and treated with cisplatin for 48 h (*n* = 3). *P < 0.05. **C** Predicted schematic of *LINC03074*-mediated mechanisms of *MDM2* translational repression (left panel) and apoptosis stimulation (right panel).

MDM2 is a major negative regulator of p53; MDM2 acts as an E3 ubiquitin ligase that recognizes p53 and acts as a transcriptional repressor of *p53* [4, 5]. To test whether *LINC03074* affected p53 protein levels and function, we performed immunoblotting using anti-p53 and anti-phosphorylated p53 (Ser15) antibodies. p53 is activated by phosphorylation in response to DNA damage, and its Ser15 residue is the major phosphorylation site [27]. Immunoblotting confirmed that cisplatin-induced DNA damage markedly increased the p53 protein levels and promoted p53 phosphorylation (Fig. 6A). Interestingly, *LINC03074* knockdown did not affect the p53 or phosphorylated p53 levels (Fig. 6A). MDM2 has been reported to interact with various proteins other than p53, and the apoptosis-related transcription factor E2F1 is one of its target proteins [28, 29]. Immunoblotting with an anti-E2F1 antibody showed that E2F1 levels were increased by cisplatin treatment and decreased by *LINC0374* knockdown, in the presence or absence of cisplatin (Fig. 6A). E2F1 induces apoptosis through several mechanisms, including activation of p53-dependent and -independent pathways and inhibition of survival signaling [30]. To elucidate the mechanism by which *LINC03074* mediates apoptosis, we examined the effects of *LINC03074* knockdown on E2F1 target gene expression. Among the apoptotic genes targeted by E2F1, *p73*, and *BIM* are transcriptionally regulated by E2F1, whereas *PUMA* and *NOXA* are regulated by E2F1 and p53 [30]. Of the four apoptosis-related genes subjected to mRNA quantification, only *p73* exhibited decreased mRNA levels following *LINC03074* knockdown with cisplatin (Fig. 6B). In addition, *p73* mRNA levels increased in response to cisplatin treatment, regardless of *LINC03074* knockdown (Fig. 6B). *BIM*, *PUMA*, and *NOXA* mRNAs showed a tendency to increase with *LINC03074* knockdown with cisplatin, but no cisplatin addition-dependent increase was observed without *LINC03074* knockdown (Fig. 6B). Our results indicate that cisplatin-induced apoptosis of seminoma cells is associated with the increased expression of *p73*. *LINC03074* contributes to the upregulation of *p73* by increasing E2F1 expression, which may indirectly affect the expression of other apoptotic genes (Fig. 6C).

## Discussion

MDM2 expression levels are associated with chemotherapy resistance in human malignancies and are regulated at multiple levels [8]. In this study, we suggest that MDM2 levels are regulated by the testis-specific lncRNA *LINC03074* during post-transcription. The *LINC03074*-MDM2-mediated apoptosis regulatory pathway provides new insights into the mechanisms of the DNA damage response in TGCT cells.

A total of 17 pairs of interaction sequences were predicted within the *Alu* element of *LINC03074* with the five *Alu* elements in the 3′UTR of *MDM2* mRNA (data from lncRRIsearch; shown in Fig. 2A). Our results suggest the following: (1) *LINC03074* binds complementarily to either *Alu* element of the *MDM2* 3′UTR via its own *Alu* element; (2) *LINC03074* modulates the binding of STAU1 and ADAR1 to the IR*Alus* of the *MDM2* 3′UTR; and (3) *LINC03074* interacts with STAU1 but not ADAR1 in the nucleus. These results suggest that *LINC03074* functions as an RNA chaperone for *MDM2* mRNA and that *LINC03074*-induced conformational changes convert the dsRNA-binding factor that binds to the 3′UTR of *MDM2*. It is speculated that ADAR1 and STAU1 competitively bind to *MDM2* mRNA because the interaction of ADAR1 with *MDM2* mRNA has been reported to suppress STAU1-MDM2 mRNA binding [16]. Therefore, we concluded that *LINC03074* binds to *MDM2* mRNA to promote STAU1 recruitment, thereby indirectly suppressing ADAR1-MDM2 mRNA binding. The intracellular molecular ratio of *LINC03074* to *MDM2* mRNA in the TCam-2 cells was approximately 1:166 (Supplementary Fig. S2). As an example of how a small amount of lncRNA can act on a large number of target molecules, previous reports have shown that *SLERT* is recycled to induce conformational changes in DDX21, which has approximately 1000 times more molecules than *SLERT* [31]. Further detailed molecular analysis is needed to determine the mechanism by which *LINC03074* exerts its action and modifies more targets than its stoichiometry. In addition, the significance of the differences in *LINC03074* levels between carcinoma and normal testis tissues needs to be investigated.

Based on the notion that protein is produced by mRNA translation, total intracellular mRNA decrease and protein increase in *MDM2* caused by *LINC03074* knockdown seem to be inconsistent (Fig. 3A, B). Besides mRNA to be translated, cytoplasmic dsRNA from viruses has been reported to elicit PKR-mediated inhibition of translation initiation [25]. PKR is activated by binding to virus-derived dsRNA and phosphorylates eukaryotic translation initiation factor 2A (eIF2A), resulting in translational repression [25]. It has been reported that downregulation of paraspeckle components, such as *NEAT1* and NONO, increases cytoplasmic 3′UTR IR*Alus* mRNAs, which increases phosphorylation of PKR and eIF2A, resulting in intracellular translational repression [23]. In this study, we demonstrated that downregulation of *LINC03074* suppresses STAU1-mediated nuclear export of 3′UTR IR*Alus* mRNA, resulting in attenuated PKR-mediated translational repression (Fig. 4). *LINC03074* had an opposite effect on the post-transcriptional regulation of IR*Alus* mRNA to the paraspeckle components, but the relationship between *LINC03074* and NONO is unclear given that no interaction between them has been found (Fig. 4A). In addition, the mechanism by which cytoplasmic *MDM2* mRNA is assigned to the opposite fates—PKR-mediated translational repression and translation initiation—remains unclear. Further investigation of the molecular mechanisms of *LINC03074*-mediated translational repression will provide new insights into the post-transcriptional regulation of the 3′UTR IR*Alus* mRNA.

The relationship between MDM2 and p53 expression in TGCTs remains unclear, although MDM2 overexpression plays an important role in suppressing p53 activity in numerous tumors that retain wild-type p53 [32, 33]. Several reports have suggested that p53 degradation by MDM2 is ineffective in TGCTs. Some studies have shown a positive correlation between MDM2 and wild-type p53 expression levels in TGCTs, while others have shown no correlation [3, 34]. We found that attenuation of MDM2 levels by *LINC03074* led to an increase in E2F1 but not p53 (Fig. 6A). E2F1 is negatively and positively regulated by MDM2 in a p53-independent manner via both direct and indirect mechanisms [28, 29]. Furthermore, E2F1 plays an important role in regulating cell proliferation and differentiation by significantly influencing cell cycle progression and survival through extensive crosstalk with p53 [35]. In this study, we demonstrated that the *p73*, a target gene of E2F1 related to apoptosis, is involved in the responsiveness of seminoma cells to cisplatin, and its expression is regulated by *LINC03074* (Fig. 6B). Considering that the MDM2-E2F1-p73 pathway is predicted to play an important role in chemotherapy resistance of TGCTs, further insights into this pathway may lead to the development of new therapies for TGCTs.

## Materials and methods

Detailed information on “Materials and methods” is shown in the Supplementary information.

### Human tumor samples

Samples of histologically normal testicular lesions and cancerous lesions were obtained from the surgical specimens of patients who underwent radical orchiectomy at the Kyoto Prefectural University of Medicine. Informed consent was obtained from all subjects. The use of surgical and autopsy specimens for molecular analysis was approved by the Institutional Ethics Committee of the hospital (Clinical trial registration no. ERB-C-2990).

### RNA immunoprecipitation

TCam-2 cells were transfected with *siLINC03074* and incubated for 72 h. Nuclear and cytoplasmic extracts were prepared as described previously (described in detail in the next section) [16]. To detect the interaction between RNA and protein, cellular lysates were incubated with anti-ADAR1 (15.8.6; Santa Cruz Biotechnology), anti-NONO (11058-1-AP; Proteintech), anti-STAU1 (C-4; Santa Cruz Biotechnology), anti-PKR (18244-1-AP; Proteintech) or anti-IgG (I5006; Sigma) antibodies at 4 °C for 18 h and then mixed with Dynabeads　Protein G (Thermo Fisher) at 4 °C for 1 h. Immunoprecipitated RNAs were isolated using ISOGEN (NIPPON GENE), and quantified via RT-qPCR, as described in Supplementary information.

### Nuclear and cytoplasmic fractionation

Nuclear and cytoplasmic fractions were obtained as previously reported, with some modifications [16]. TCam-2 cells were lysed with the nuclear fractionation buffer (10 mM Tris-HCl, pH 7.5, 10 mM NaCl, 0.2% NP-40, 3 mM MgCl_2_, 100 U/ml RNase Inhibitor) at 4 °C for 10 min and centrifuged at 13000 rpm at 4 °C for 10 min. The supernatant was used as the cytoplasmic fraction. The pellet was washed with the nuclear fractionation buffer and centrifuged at 13000 rpm at 4 °C for 10 min. The pellet was used as the nuclear fraction. The respective markers of the nuclear and cytoplasmic fractions, *5S-rRNA* and *GAPDH*, respectively, were used as controls.

### Statistical analysis

Statistical analyses were performed using *t*-tests or ANOVA, as appropriate. Statistical significance was set at *P* < 0.05. Each experiment was repeated at least three times. Information on statistical measures is provided in the legend of each figure.

### Supplementary information

Supporting Information

raw data

## Data Availability

All data of this manuscript are included in the main text and supplementary files.
