# Supplementary material for: A testis-specific lncRNA functions as a post-transcriptional regulator of MDM2 and stimulates apoptosis of testicular germ cell tumor cells
Source: Cell Death Discov. 2024 Aug 3;10:348. doi: 10.1038/s41420-024-02119-8 (PMC11297958; doi:10.1038/s41420-024-02119-8)
Supplement: Supplementary file 1 — Supporting Information [file 41420_2024_2119_MOESM1_ESM.docx]

**Supplemental Material for**

**A testis-specific lncRNA functions as a post-transcriptional regulator of *MDM2* and stimulates apoptosis of testicular germ cell tumor cells**

**Saya Ito ^# *^, Akihisa Ueno ^*^, Takashi Ueda, Ryota Ogura, Satoshi Sako, Yusuke Gabata, Osamu Ukimura**

Department of Urology, Graduate School of Medical Science, Kyoto Prefectural University of Medicine, Kyoto-City, Kyoto, Japan.

^*^ These authors contributed equally to this work

**^#^ Corresponding author:** Saya Ito, Department of Urology, Graduate School of Medical Science, Kyoto Prefectural University of Medicine, 465 Kajii-cho, Kawaramachi-Hirokoji, Kamigyo-ku, Kyoto 602-8566, Japan.

E-mail: [**itosaya@koto.kpu-m.ac.jp**](mailto:itosaya@koto.kpu-m.ac.jp)

**This file includes:**

Supplementary Material and Methods

Supplementary References

Figures. S1 to S5

Tables. S1 and S2

**Supplementary Material and Methods**

**RNA *in situ* hybridization (ISH)**

RNA ISH was performed according to the standard method using paraffin-embedded testis tissue sections (T2234260 and T2235260; BioChain). Sections were deparaffinized, treated with 5 µg/mL proteinase K at 37 °C for 30 min, acetylated, and prehybridized with hybridization buffer [5× SSC (0.75 M NaCl and 0.075 M sodium citrate), 2% blocking reagents (Roche), 50% formamide, 0.1% N-lauroylsarcosine, and 0.1% SDS] at 60 °C for 1 h. Hybridization was performed overnight at 60 °C with DIG-labeled probes for sense or antisense *LINC03074* in hybridization buffer. DIG-labeled RNA probes for *LINC03074* were generated by *in vitro* transcription of linearized DNA templates using either T7 or Sp6 RNA polymerases (Takara) in the presence of DIG-UTP (Roche). After hybridization, sections were washed two times for 15 min each at 60 °C in 50% formamide containing 2× SSC (0.3 M NaCl and 0.03 M sodium citrate) and treated 20 μg/mL RNaseA in RNase buffer [0.5 M NaCl, 10 mM Tris-HCl (pH 8.0), and 1 mM EDTA] for 30 min at 37 °C. Sections were washed twice with 2× SSC containing 0.1% N-lauroylsarcosine at 37 °C for 15 min each and then incubated in blocking buffer [1% blocking reagent, 0.1 M Tris-HCl (pH 7.5), and 0.15 M NaCl] at 25 °C for 30 min. Next, the sections were incubated with alkaline phosphatase-conjugated anti-DIG Fab fragments (1:1000 dilution; Roche) overnight at 4 °C and visualized with BCIP-NBT substrate solution (Nacalai Tesque) by incubating overnight 25 °C. Following ISH, the sections were counterstained with Nuclear Fast Red (Vector Laboratories) and mounted using a mounting medium (Entellan new, Merck). All images were obtained using the AdvanView imaging software included in the AdvanCam-E3Rs/ALL-IN-ONE system (AdvanVision).

**Cell culture and transfection**

HEK293 cells were provided by the RIKEN BioResource Research Center through the National BioResource Project of the Ministry for Education, Culture Sports, Science and Technology (MEXT), Japan, and were cultured in Dulbecco’s modified Eagle’s medium (DMEM, Nacalai Tesque) with 10% fetal bovine serum (FBS) at 37 °C under 5% CO_2_. TCam-2 cell lines were kindly provided by Dr. R. Kitazawa [1]. NCCIT, NEC8, and NEC14 cell lines were purchased from ATCC. TCam-2, NCCIT, NEC8, and NEC14 cells were cultured in RPMI 1640 medium (Nacalai Tesque) with 10% FBS at 37 °C under 5% CO_2_. Cisplatin (Fuji Film) was added to the culture medium at concentrations ranging from 1 to 50 μM. PKR inhibitor (Cayman chemical company) was added to the culture medium at a final concentration of 1 μM and incubated for 24 h.

To knockdown *LINC03074* or STAU1 expression, cells were transfected with either an siRNA against *LINC03074* (SI05737032, SI05737039, SI05737046, Qiagen; *siLINC03074*), STAU1 (s13546, s13548, ambion; *siSTAU1*) or a negative control siRNA (452002, Invitrogen; *siControl*) using Lipofectamine RNAiMAX (Invitrogen) for more than 24 h. Most experiments were performed with *siLINC03074*_#3 (SI05737046), which has the highest knockdown efficiency, as shown in Fig. 3A. Transfection of expression plasmids into cells was performed using Lipofectamine 3000 (Invitrogen) in an antibiotic-free medium for 24 h.

Expression plasmids were prepared as follows: *hMDM2* [Ensemble Transcript ID: ENST00000258149.11; 3' UTR (full length, FL): 1795–7490 bp, 3' UTR (Δ5’-Alu): 3400–7490 bp, coding sequence (CDS): 302–1794 bp, CDS + 3' UTR (FL): 302–7490 bp, CDS + 3' UTR (5’-ΔAlu): deletion of 1795–3400 bp of CDS + 3' UTR (FL)] and *LINC03074* [Ensemble Transcript ID: ENST00000515329.1; FL: 1–2652 bp] were amplified using the HEK293 cell genome as template, specific primers, and PrimeSTAR MAX DNA polymerase (Takara); they were cloned into the pcDNA3 vector (Invitrogen) using In-Fusion HD Cloning Kit (Takara). For *MDM2* cloning, pcDNA3 vector with FLAG tag inserted at BamHI/EcoRI site was used. Furthermore, expression plasmids for *LINC03074* [ΔAlu: deletion of 1238–1599 bp of FL] were produced by mutagenesis PCR using expression plasmid for *LINC03074* (FL) as templates. The primer sets are listed in Table S2.

**Capture hybridization analysis of RNA targets (CHART) assay**

CHART assays were performed as previously reported, with some modifications [2]. Harvested TCam-2 cells were fixed with 1 % formaldehyde at 25 °C for 10 min. Cell pellets were washed with PBS, suspended in sucrose buffer [0.3 M sucrose, 1 % Triton X-100, 10 mM HEPES (pH 7.5), 100 mM potassium acetate, 0.1 mM EGTA, 0.5 mM spermidine, 0.15 mM spermine, 1mM DTT, 20 U/ml RNase Inhibitor (TOYOBO)] and homogenized twenty times using ice-cold Dounce homogenizer. Glycerol buffer (25 % glycerol, 10 mM HEPES, 1 mM EDTA, 0.1 mM EGTA, 100 mM potassium acetate, 0.5 mM spermidine, 0.15 mM spermine, 1 mM DTT, 10 U/ml RNase Inhibitor) was added and centrifuged at 1000 x *g* at 4 °C for 10 min. Nuclear pellets were resuspended in 3.2 % formaldehyde with PBS and shaken at 25 °C for 30 min. After washing with PBS and centrifuge for 5 min at 1000 x *g*, pellets were resuspended in 0.5 ml of Wash buffer 100 [100 mM NaCl, 10 mM HEPES (pH 7.5), 2 mM EDTA, 1 mM EGTA, 0.2 % SDS, 0.1 % N-lauroylsarcosine] and sonicated using an ultrasonic generator (UR-21P, TOMY) at 4 °C (power level 6, 15 sec x 10). After centrifugation for 20 min at 16000 x *g* at 4 °C, 0.05 ml of the supernatant was mixed with 0.1 ml of hybridization buffer [20 mM HEPES (pH 7.5), 817 mM NaCl, 1.9 M urea, 0.4% SDS, 5.7 mM EDTA, 0.3 mM EGTA, 0.03% sodium deoxycholate, and Denhardt solution] and incubated with C-oligos (final concentration: 570 nM, 5'-CCAAAAGTGGGTGGGTGTAGCATG-C18 spacer-BioTEG-3', IDT) at 25 °C for 8 h. MyOne C1 streptavidin beads (Invitrogen) were added to binding reaction and incubated at 25 °C for 18 h. After five washes with WB250 [250 mM NaCl, 10 mM HEPES (pH 7.5), 2 mM EDTA, 1 mM EGTA, 0.2% SDS, and 0.1% N-lauroylsarcosine], each binding reaction was incubated with 4 mg/mL of biotin (Nacalai Tesque) in WB250 at 25 °C for 1 h and then centrifuged briefly. The supernatant was reacted with a solution consisting of 200 mM Tris-HCl (pH 7.5), 2% SDS, and 5 mg/mL Proteinase K at 55 °C for 1 h and then at 65 °C for 1 h. The mixture was incubated with 0.1 U/mL DNaseI (Takara) at 25 °C for 30 min. RNA was purified using PureLink RNA Mini kit (Invitrogen) and quantified using RT-qPCR, as described below. The primer sets used for PCR are listed in Table S1.

***In vitro* RNA pull-down assays**

*In vitro,* RNA pull-down assays were performed using Dynabeads M-280 Streptavidin (Invitrogen), according to the manufacturer’s instructions, with biotin-labeled *MDM2* 3' UTR and *LINC03074*. Biotin-labeled *MDM2* 3' UTR (FL or Δ5’-Alu) was transcribed *in vitro* using a Biotin Labeling Mix (Roche), T7 RNA polymerase (Takara), and a linearized plasmid DNA (*MDM2* 3' UTR*-pcDNA3*). Transcribed RNAs were purified using a PureLink RNA Mini kit (Ambion) and confirmed via RT-qPCR (data not shown). RNAs (1 μg) were heated to 90 °C for 2 min, transferred to ice for 2 min, and shifted to 25 °C for 20 min in RNA structure buffer [10 mM Tris-HCl (pH 7.0), 0.1 M KCl, and 10 mM MgCl_2_]. Folded RNA was incubated at 25 °C for 30 min with streptavidin beads in DEPC-treated 0.1 M NaCl and washed three times with washing buffer [5 mM Tris-HCl (pH 7.5), 0.5 mM EDTA, and 1 M NaCl]. In addition, total RNA was isolated from *LINC03074* (FL or ΔAlu) overexpressing HEK293 cells previously transfected with expression plasmids for 24 h using ISOGEN reagent (Wako). Total RNA (5 μg) was added to each binding reaction, incubated at 25 °C for 15 min, and washed with washing buffer. After 3 washes, each binding reaction was eluted from streptavidin beads by incubation in elution buffer (10 mM EDTA in formamide) at 90 °C for 3 min and quantified using RT-qPCR as described below. The primer sets used for PCR are listed in Table S1.

**RT-qPCR**

RT-qPCR was performed according to the manufacturer’s instructions. Briefly, total RNA was isolated using an ISOGEN kit, and reverse transcription (RT) was performed using PrimeScript RT Master Mix (Takara). cDNAs were quantified via RT-qPCR using the SYBR qPCR mix (Toyobo) and a Thermal Cycler TP800 (Takara). The primer sets used are listed in Table S1.

**Western blotting**

Whole-cell lysates were extracted with lysis buffer [10 mM Tris-HCl (pH 7.8), 1% NP-40, 0.15 M NaCl, and 1 mM EDTA]. Western blotting was performed using standard methods [3]. Band intensity was quantified using Image Lab 6.1 (BioRad). The commercially available antibodies used included the following: anti-MDM2 (SMP14; Santa Cruz Biotechnology), anti-p53 (DO-1; Santa Cruz Biotechnology), anti-phospho-p53-S15 (9284; Cell Signaling Technology), anti-E2F1 (KH95; Santa Cruz Biotechnology), anti-β-actin (GTX 109639; GeneTex), and anti-flag (F7425; Sigma). Band intensity was quantified by Image Lab 6.1. software (BIO-RAD). The measured values were first compensated for β-Actin protein levels, and then the relative values to each control were calculated.

**Cell growth assay**

TCam-2 and NEC8 cells were transfected with *siLINC03074* and incubated for 6 h. Transfected cells were plated in 96-well plates at 1500 cells per well and treated with cisplatin (at final concentrations of 0, 1, 10, and 50 mM). Live cells were counted using the Kit-8 cell counting method (Dojindo).

**Apoptosis assay**

Cells were transfected with *siLINC03074* and treated with 20 μM cisplatin. After 48 h of incubation, the cells were stained with Annexin V-FITC (MBL) and propidium iodide (Merck) according to the manufacturer’s instructions. Apoptotic cells were detected using flow cytometry (BD FACSCanto II).

**Cell cycle assay**

Cells were transfected with *siLINC03074* and treated with 20 μM cisplatin. After 48 h of incubation, cells were fixed with 70% ethanol on ice for 30 min and stained with propidium iodide, according to the manufacturer’s instructions. The DNA content was quantified using flow cytometry.

**Supplementary References**
1. Mizuno Y, Gotoh A, Kamidono S, Kitazawa S. [Establishment and characterization of a new human testicular germ cell tumor cell line (TCam-2)]. Nihon Hinyokika Gakkai Zasshi. 1993; 84: 1211–1218. doi: [10.5980/jpnjurol1989.84.1211](https://doi.org/10.5980/jpnjurol1989.84.1211), PubMed PMID: [8394948](http://www.ncbi.nlm.nih.gov/pubmed/8394948).

2. Simon MD, Wang CI, Kharchenko PV, West JA, Chapman BA, Alekseyenko AA, et al. The genomic binding sites of a noncoding RNA. Proc Natl Acad Sci U S A. 2011; 108: 20497–20502. Epub 20111205. doi: [10.1073/pnas.1113536108](https://doi.org/10.1073/pnas.1113536108), PubMed PMID: [22143764](http://www.ncbi.nlm.nih.gov/pubmed/22143764), PubMed Central PMCID: [PMC3251105](https://www.ncbi.nlm.nih.gov/pmc/articles/PMC3251105).

3. Ito S, Fujiyama-Nakamura S, Kimura S, Lim J, Kamoshida Y, Shiozaki-Sato Y, et al. Epigenetic silencing of core histone genes by HERS in Drosophila. Mol Cell. 2012; 45: 494–504. doi: [10.1016/j.molcel.2011.12.029](https://doi.org/10.1016/j.molcel.2011.12.029), PubMed PMID: [22365829](http://www.ncbi.nlm.nih.gov/pubmed/22365829)

**Supplementary Figures**


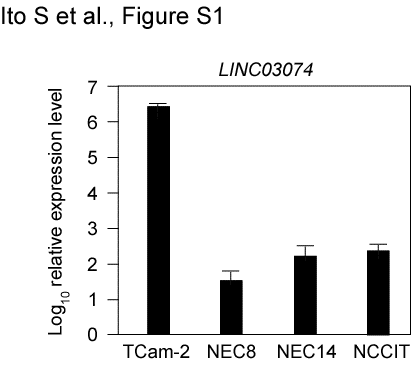


**Figure S1.** *LINC03074* expression in cultured cells derived from testicular germ cell tumors. The relative expression levels of *LINC03074* and *GAPDH* were measured using RT-qPCR. TCam-2 is a seminoma cell line, whereas NEC8, NEC14, and NCCIT are nonseminoma cell lines.


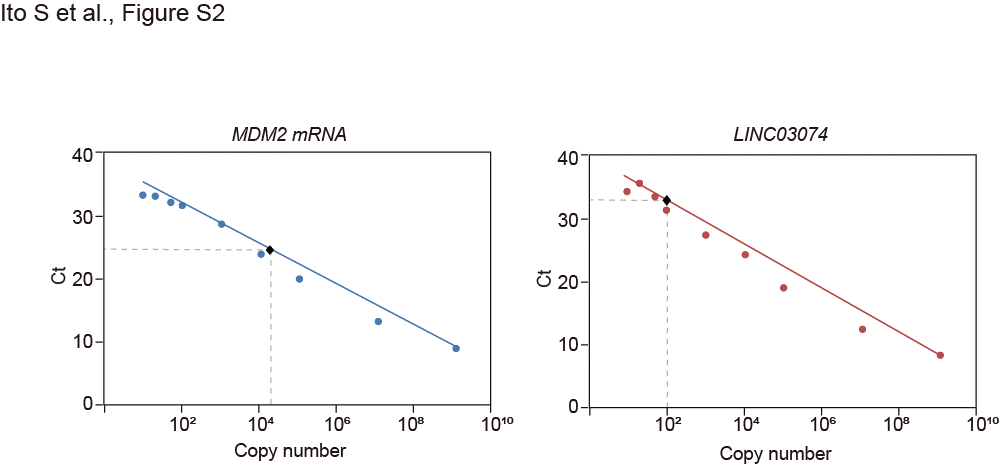


**Figure S2.** Quantification of intracellular RNA molecules in *MDM2* and *LINC034074*.

RNA copy number of *MDM2* and *LINC03074* was quantified with RT-qPCR using 1 ng of total RNA extracted from TCam-2 cells. Calculated according to the Ct value of the calibration curve (lines) with standard cDNAs (round dots), there were 10220 copies of *MDM2* mRNA and 122 copies of *LINC03074* in 1 ng of total RNA (diamond-shaped black dots). The molecular ratio of *LINC03074* to *MDM2* mRNA in TCam-2 cells is approximately 1:166. Assuming 10 pg of total RNA per cell, the RNA molecules of *LINC03074* and *MDM2* per cell are estimated to be about 1.2 and 203, respectively.


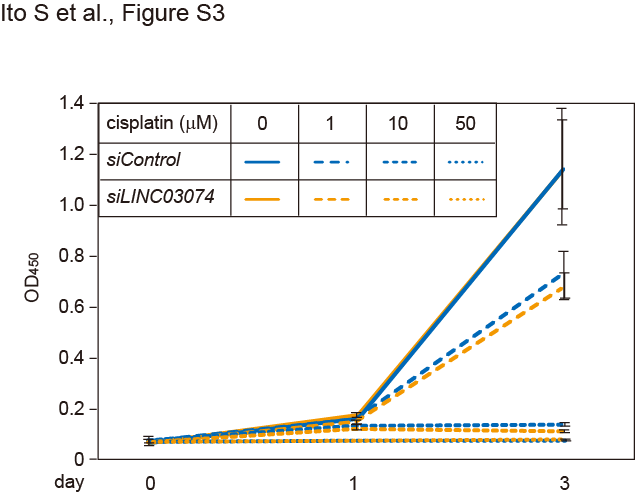


**Figure S3.** Proliferation of NEC8 cells**.**

Cell growth assay of NEC8 cells transfected with an siRNA for *LINC03074* (*siLINC03074*) and treated with different concentrations of cisplatin*.* Absorbance at 450 nm (OD_450_) was used to estimate cell concentration. Data represent the means ± SEM (n = 3).


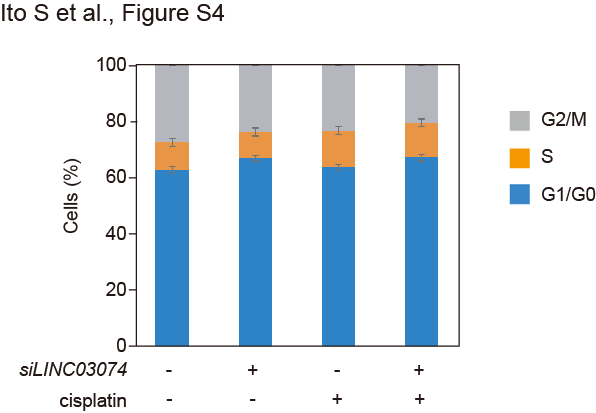


**Figure S4.** Cell cycle of *LINC03074*-knockdown TCam-2 cells treated with cisplatin. TCam-2 cells were transfected with an siRNA for *LINC03074* (*siLINC03074*) and treated with 20 μM cisplatin for 48 h. Data represent the means ± SEM (n = 3).


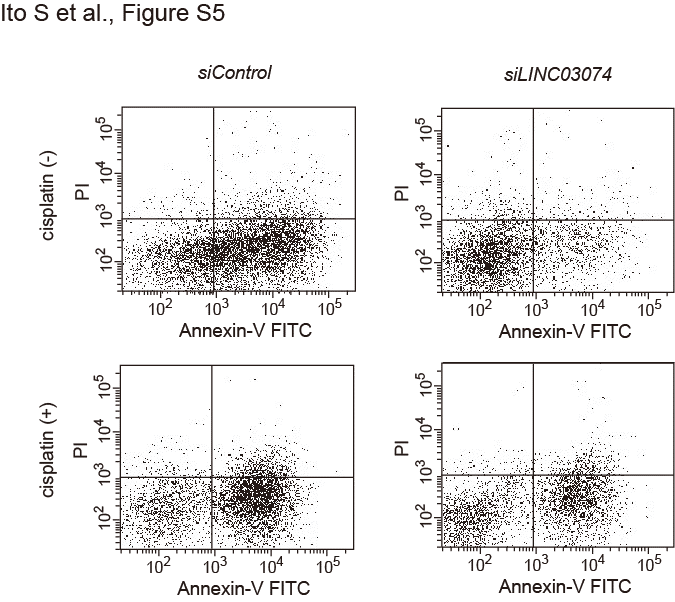
**Figure S5.** Representative images of each group of apoptosis assays using *LINC03074* knockdown TCam-2 cells treated with cisplatin.

**Supplementary Tables**

**Table S1.** Primer sets for qPCR analysis.

| Gene |  | Sequence |
| --- | --- | --- |
| *LINC03074* | forward | 5'-AAGCAGAGAGGTCAGAATTCAAACTCAGATTTCTG-3′ |
|  | reverse | 5-CAAAAGTGGGTGGGTGTAGC-3′ |
| *MDM2* | forward | 5-TGCCAAGCTTCTCTGTGAA-3′ |
|  | reverse | 5'-CGATGATTCCTGCTGATTGA-3′ |
| *18S-rRNA* | forward | 5'-GCTTAATTTGACTCAACACGGGA-3′ |
|  | reverse | 5'-AGCTATCAATCTGTCAATCCTGTC-3′ |
| *5S-rRNA* | forward | 5'-GGCCATACCACCCTGAACGC-3′ |
|  | reverse | 5'-CAGCACCCGGTATTCCCAGG-3′ |
| *GAPDH* | forward | 5-ACCACAGTCCATGCCATCAC-3′ |
|  | reverse | 5-TCCACCACCCTGTTGCTGTA-3′ |
| *p73* | forward | 5'-CATGGAGACGAGGACACGTACT -3′ |
|  | reverse | 5'-TGCCGATAGGAGTCCACCAGTG-3′ |
| *BIM* | forward | 5'-CAAGAGTTGCGGCGTATTGGAG-3′ |
|  | reverse | 5'-ACACCAGGCGGACAATGTAACG -3′ |
| *PUMA* | forward | 5'-ACCATCTCAGGAAAGGCTGT-3′ |
|  | reverse | 5-TGGCTCATTTGCTCTTCACG-3′ |
| *NOXA* | forward | 5'-ACTCTTCTGCTCAGGAACCT-3′ |
|  | reverse | 5-TGCACCTTCACATTCCTCTCA-3′ |

**Table S2.** Primer sets for cloning of expression plasmid.

|  | primer name | sequence |
| --- | --- | --- |
| *MDM2 3' UTR (FL)* | XhoI-1795-For | 5'-CACAGTGGCGGCCGCTCGAGTTGACCTGTCTATAAGAG-3' |
|  | 3415-Rev | 5'-ATAACATCATTACTCCCATCCCTTACTATGGTT-3' |
|  | 3400-For | 5'-GAGTAATGATGTTATCTGTGAAAATAGCCACCA-3' |
|  | 5535-Rev | 5'-TTCACATATGGGAGAAAAACACTAAATCAAGAT-3' |
|  | 5521-For | 5'-TCTCCCATATGTGAATTGTATATACTTAGGTGA-3' |
|  | AgeI-7490-Rev | 5'-GGTGATGATGACCGGTAGACAATCAACTATGGTT-3' |
| *MDM2 3' UTR (Δ5’-Alu)* | XhoI-3401-For | 5'-CACAGTGGCGGCCGCTCGAGGAGTAATGATGTTATCTG-3' |
|  | AgeI-7490-Rev | 5'-GGTGATGATGACCGGTAGACAATCAACTATGGTT-3' |
| *MDM2 CDS* | EcoRI-302-For | 5'-GGGGAATTCATGGTGAGGAGCAGGCAATG-3' |
|  | Xho-1794-Rev | 5'-GGGACTCGAGCTAGGGGAAATAAGTTAG-3′ |
| *LINC03074 FL* | HIndIII-1-For | 5'-TGGCTAGTTAAGCTTACTTGCTTGGGGGGAACCA-3′ |
|  | 271-Rev | 5'-TCCTCACAGTTTTCGTGAGTCATGAATGTGGGA-3′ |
|  | 257-For | 5'-CGAAAACTGTGAGGACACATGGTGAATAGAGGGCT-3′ |
|  | 1196-Rev | 5'-ACTACCCCACTCTGCAGTGTACTTTCTGCATGA-3′ |
|  | 1182-For | 5'-GCAGAGTGGGGTAGTAACTAACAAAATATGGCC-3′ |
|  | 2666-XhoI-Rev | 5'-CCCTCTAGACTCGAGGCTAAATCTCATTAACTTCC-3′ |
| *LINC03074 ΔAlu* | ΔAlu-For | 5′-GAACCAGGTCTGAGTGCTAAAACCTT-3′ |
|  | ΔAlu-Rev | 5′-CTCTATAGTCCTAAAGGTAGATCAT-3′ |
