# Supplementary material for: A testis-specific lncRNA functions as a post-transcriptional regulator of MDM2 and stimulates apoptosis of testicular germ cell tumor cells
Source: Cell Death Discov. 2024 Aug 3;10:348. doi: 10.1038/s41420-024-02119-8 (PMC11297958; doi:10.1038/s41420-024-02119-8)
Supplement: Supplementary file 2 — raw data [file 41420_2024_2119_MOESM2_ESM.docx]

**Supporting Information**

Original Blots

Figure 3B


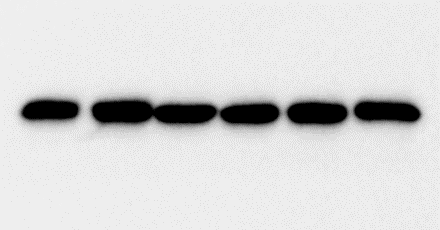

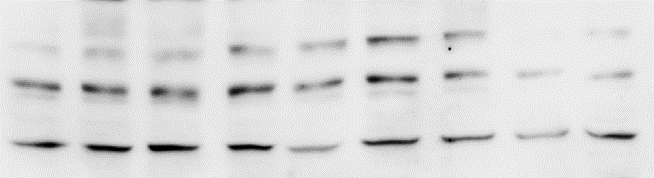

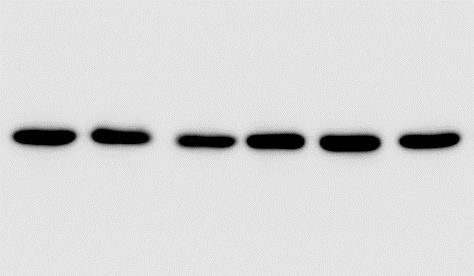
**
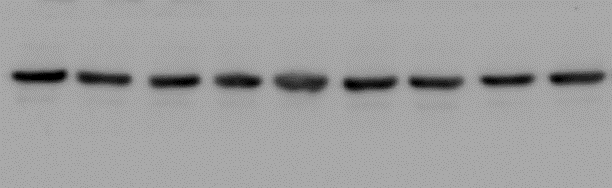
**
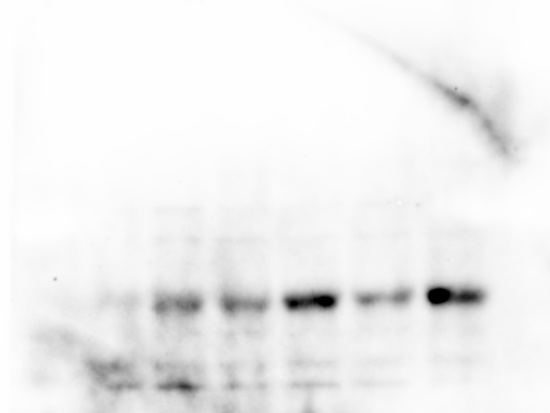

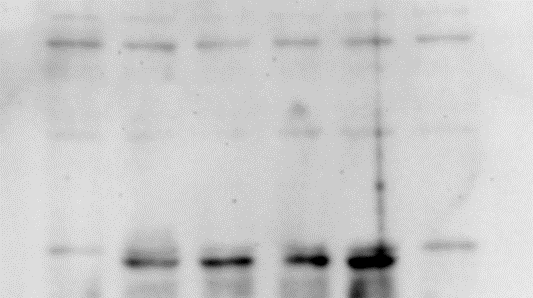


β-Actin

FLAG

β-Actin

Figure 3D

β-Actin

MDM2

Figure 4D

MDM2


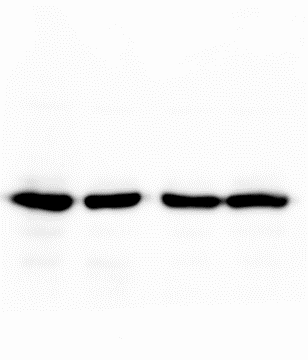

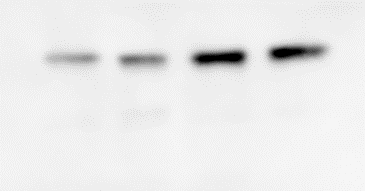

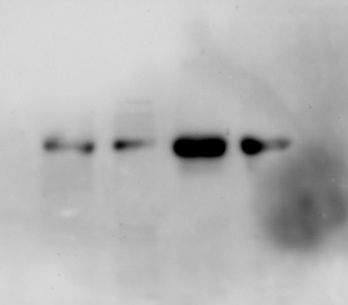

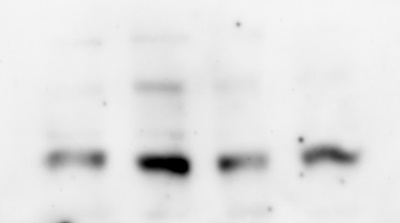

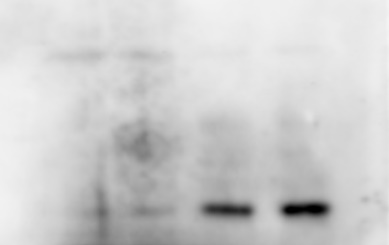


E2F1

p53

MDM2

β-Actin

P-p53

Figure 6A
